# Measures of both perceived general and specific risks and benefits differentially predict adolescent and young adult tobacco and marijuana use: findings from a Prospective Cohort Study

**DOI:** 10.1057/s41599-021-00765-2

**Published:** 2021-03-29

**Authors:** Karma McKelvey, Shivani Mathur Gaiha, Kevin L. Delucchi, Bonnie Halpern-Felsher

**Affiliations:** 1 Department of Pediatrics, School of Medicine, Stanford University, 770 Welch Road, Suite 100, Palo Alto, CA 94304, USA.; 2 Department of Psychiatry, University of California San Francisco, San Francisco, CA 94143, USA.; 3 These authors contributed equally: Karma McKelvey, Shivani Mathur Gaiha.

## Abstract

Health behavior theorists and prevention researchers use a variety of measures of adolescent and young adult (AYA) risk and benefit perceptions to predict tobacco-use and marijuana-use behaviors. However, studies have not examined whether and how perception measures that ask about likelihood of more general outcomes such as “harm” versus ask about specific risk or benefit outcomes compare or whether they differentially predict AYA willingness to use if one of your best friends were to offer it and intentions to use in the next year; and if these measures have differential ability to predict actual use of tobacco and marijuana. We used data from a prospective cohort of California AYAs to create and test new scales to measure perceptions of specific health and social outcomes related to risks (e.g., smell bad) and benefits (e.g., look cool) related to tobacco and marijuana, and then addressed three questions: (1) Whether and how measures of perceptions of specific social and health risks and benefits (for our purposes “specific measures”) and measures of perceived general harm are differentially associated with measures of willingness, social norms, and intentions to use? (2) Are specific versus general measures differentially associated with and predictive of tobacco and cannabis use behavior? (3) Are specific perceptions measures differentially predictive of behavior compared to measures of willingness, social norms, and behavioral intentions? Our results demonstrate that to better predict AYA tobacco and marijuana use, measures that address general outcomes, such as harmfulness, as well as willingness and behavioral intention should be used. We also found that measures of specific perceived risks (short-term, long-term, social) and benefits were unrelated and correlated differently with different products. For example, adolescents perceived both risks and benefits from using products like e-cigarettes, and perceived greater risk from smokeless tobacco compared to combustible cigarettes. These findings indicate that measures of specific perceived social and health outcomes can be useful to discern nuanced differences in motivation for using different substances. Study implications are important for survey dimension-reduction and assessing relationships among perceptions, motivations, and use of tobacco and marijuana products.

## Introduction

Explanations for adolescent and young adult (AYA) tobacco and marijuana use reside in theories of health behavior, which argue that behavior is influenced by risk and benefit perceptions, social norms (e.g., perceived prevalence), and willingness and intentions to engage in such behavior (Ajzen, 1985, 1991; Liu et al., 2017; Prochaska et al., 1992; Rosenstock, 1974; Smith et al., 2007). Cross-sectional and longitudinal studies have established a relationship between risk and benefit perceptions, behavioral intentions, and actual use of tobacco (i.e., cigarettes, e-cigarettes, cigars, smokeless to and hookah) and marijuana products (i.e., blunts, smoked marijuana [not blunts], and vaped marijuana) among AYAs (Bold et al., 2016, 2016; Bunnell et al., 2015; Cengelli et al., 2012; Durkin et al., 2020; Ennett et al., 2010; Morrell et al., 2010; Owotomo et al., 2018; Preventing Tobacco Use Among Youth and Young Adults, 2012; Roditis, Lee et al., 2016; Roditis et al., 2016). Despite the important theoretical and empirical relationship between perceptions of risks and benefits and actual tobacco and marijuana use, the measurement of such perceptions varies greatly across studies, including differences in how the questions are asked and outcomes being assessed (Kaufman et al., 2020). One common difference in perceptions measures used in AYA tobacco-related or marijuana-related research is whether participants are asked about perceptions of general harm related to tobacco or marijuana (e.g., rate your perception of harmfulness to health) (Roditis et al., 2016) or about perceptions of specific health and social outcomes (e.g., short-term health outcomes such as shortness of breath, long-term outcomes such as lung cancer, or social risks such as getting into trouble) or perceptions of specific benefits (e.g., looking cool or more mature, fitting in with peers) (Bold et al., 2016; Durkin et al., 2020; Kaufman et al., 2002; Morrell et al., 2010; Owotomo et al., 2018; Preventing Tobacco Use Among Youth and Young Adults, 2012; Roditis, Lee et al. 2016; Slovic, 2000; Song et al., 2009). Studies have independently used measures assessing perceptions of both general and specific risks related to tobacco and marijuana use to predict actual use (Orlan et al., 2019; Slovic, 2000; Song et al., 2009; Strong et al., 2019).

Absent in the literature is an assessment of the psychometric properties of measures assessing perceptions of specific risks and benefits. Further, studies have not examined whether and how perceptions measures that ask about a more general outcome such as “harm” versus specific outcomes related to risks or benefits compare with AYA’s willingness to use if one of your best friends were to offer it (Centers for Disease Control and Prevention, 2019; Orlan et al., 2019) and intentions to use in the next year (Centers for Disease Control and Prevention, 2019); and if they have differential ability to predict actual use of tobacco and marijuana. To improve measurement utility and support measurement uniformity in the field of risk and benefit perceptions, and to provide clear direction for the development of more parsimonious perception surveys, we refined and validated perceptions measurement scales composed of items asking about short-term social and health risks, long-term health risks, and benefits. We then set out to answer the following three questions regarding AYA use of tobacco (cigarettes, e-cigarettes, cigars, smokeless tobacco, and hookah), blunts (hollowed out cigars filled with marijuana), smoked marijuana (not including blunts), and vaped marijuana: (1) Whether and how measures of perceptions of specific social and health risks and benefits (for our purposes “specific measures”) and measures of perceived general harm are differentially associated with measures of willingness, social norms, and intentions to use? (2) Are specific versus general measures differentially associated with and predictive of behavior? (3) Are specific perceptions measures differentially predictive of behavior compared to measures of willingness, social norms, and behavioral intentions?

Findings from this study will identify measures of perceived risks and benefits most strongly linked with intentions, willingness, and actual behavior. Ultimately, the findings will support uniformity across studies and improve study comparability, thus increasing generalizability of findings and enabling a cohesive evidence-base to understand and accurately predict AYA tobacco and marijuana use behaviors.

## Methods

### Data source.

Data for this study came from the Tobacco Perceptions Study, an 8-wave prospective cohort study designed to measure tobacco and marijuana perceptions, intentions, actual use, social norms, and marketing among California high school students. The 10 high schools were chosen using convenience sampling; the original sampling frame included all students in the 9th and 12th grades from these schools. Ninth graders were chosen since the average age of first trying a cigarette in the U.S. was 14.5 years, thus providing for a prospective examination of the impact of perceptions on tobacco use. Twelfth graders were chosen as following them into young adulthood would afford a broader sample of young adults then obtained by simply sampling college students or those who joined the workforce. Our cohort study was designed to examine changes in use and perceptions of tobacco products over time instead of making population-level estimates, which was suitable to test the validity, reliability, and predictive strength of the measurement items used. Independent variables came from Wave 1 through Wave 3 and dependent variables from Waves 5–7. Details of the study design and procedures are provided elsewhere (Gorukanti et al., 2017; Lin et al., 2020; McKelvey, Baiocchi et al., 2018; McKelvey, Popova et al., 2018; Roditis et al., 2016). Variables tested are described in detail below.

### Procedure.

Consented participants received an email containing a link to each survey, administered through Qualtrics, Qualtrics Labs; Provo, UT. Data were collected between 2014 and 2019, as follows: Wave 1 survey was administered from July 13, 2014–October 11, 2015; Wave 2 from July 22, 2015–March 30, 2016; Wave 3 from June 9, 2016–September 22, 2016; Wave 4 from March 16, 2017–July 4, 2017; Wave 5 from October 23, 2017–April 9, 2018; Wave 6 from April 6, 2018–August 17, 2018; Wave 7 from August 27, 2018–November 11, 2018; and Wave 8 from January 22, 2019 to April 14, 2019. Participants who completed the baseline survey received a $10 gift card, and the gift card amount increased by $5 with each subsequent survey wave. Stanford University’s Institutional Review Board approved this study. Informed consent was obtained from all participants 18 years and above and legal guardians of participants under 18 years; and assent was obtained from all participants under age 18.

### Participants.

There were 1299 students recruited and consented for the baseline survey in 2014–15. Of the *N* = 772 who completed the baseline survey, *n* = 486 (63%) reported female sex and the mean age was 16.01 years (SD = 2.00). The sample was ethnically diverse and consisted of participants who identified as: Hispanic (*n* = 283, 37%), White, not-Hispanic (*n* = 204, 26%), Asian/Pacific Islander (*n* = 171, 22%), and other (*n* = 114, 15%). Participants reported ever-use of different marijuana and tobacco products, as follows: marijuana (not including blunts) (*n* = 195, 25%); hookah (165, 21%); e-cigarettes (146, 19%); blunts (142, 18%); cigarettes (98, 13%); cigars (48, 6 %); and smokeless tobacco (19, 4%). Overall, 57% of participants who completed the baseline survey in Wave 1 went on to complete surveys in Wave 7 (*n* = 408).

### Measures

#### Demographics.

Participants provided information including school, age, sex, and race/ethnicity. A priori it was decided to not covary sex and ethnicity as previous work has shown no association between these variables and outcomes of interest (Collins et al., 2017; Gorukanti et al., 2017; Weaver et al., 2018; Yingst et al., 2019).

#### Specific measures of perceived risks and benefits associated with tobacco and marijuana use

##### Short-term risks

Participants read: “Whether or not you have used any of the products, imagine that you just began using one of the products [listed products: cigarettes, e-cigarettes, cigars, smokeless tobacco, hookah, blunts, smoked marijuana, vaped marijuana] below. You use it about 2–3 times a day, every day. Sometimes you use it alone and sometimes you use it with friends.” Participants then indicated the percent chance (from 0% to 100%) of: (a) becoming addicted, (b) being able to quit whenever they want, (c) still using the product in 5 years, (d) feeling jittery/nervous, (e) having a bad cough, (f) suffering from more colds, (g) having trouble catching their breath, (h) developing mouth sores, (i) having worse performance in sports, (j) friends being upset with them, (k) feeling high or buzzed, (l) getting in trouble, and (m) having bad breath. For cigars, cigarettes, smokeless, and e-cigarettes, scales were created using these items from Wave 1; for hookah, blunts, smoked marijuana, and vaped marijuana, scales were created using these measures from Wave 3 (which was the earliest wave available).

##### Long-term risks

Participants read: “Imagine now that you continue to use one of the products [listed products: cigarettes, e-cigarettes, cigars, smokeless tobacco, hookah, blunts, smoked marijuana, vaped marijuana] below 2–3 times a day, every day for the rest of your life.” They were then asked to indicate the percent chance (from 0% to 100%) of: (a) developing oral cancer, (b) getting wrinkles, (c) having a heart attack, (d) developing lung cancer, (e) developing another tobacco-related illness, and (f) death from a tobacco-related illness. For cigars, cigarettes, smokeless, and e-cigarettes, scales were created using these items from Wave 1; for hookah, blunts, smoked marijuana, and vaped marijuana, scales were created using these measures from Wave 3 (which was the earliest wave available).

##### Benefits

Given the same scenario and list of products as above, participants indicated the percent chance (from 0% to 100%) of: (a) having better concentration, (b) feeling less stressed, (c) feeling high or buzzed, (d) being less hungry, (e) looking cool, (f) looking more mature, and(g) fitting in with their peers. In the creation of our scales, these seven items together with the 13 listed above in *Short-Term Risks* were combined and factor analyzed as described more fully below. For cigars, cigarettes, smokeless, and e-cigarettes, scales were created using these items from Wave 1; for hookah, blunts, smoked marijuana, and vaped marijuana, scales were created using these measures from Wave 3 (which was the earliest wave available).

#### General measures of perceived risks associated with tobacco-and marijuana use.

For all products at Wave 1 as listed above, participants read: “Imagine you use the products below 2–3 times a day, every day. How harmful would this be for your health?” and chose among the following responses: 1 = not at all harmful, 2 = slightly harmful, 3 = moderately harmful, 4 = quite harmful, 5 = extremely harmful.

#### Behavioral intentions.

Participants answered the following question in Wave 1 for cigarettes, cigars, and e-cigarettes: How likely is it that over the next 6 months you will try [product] for the first time? Responses ranged from 1 to 5 (1 = already tried, 2 = very unlikely, 3 = somewhat unlikely, 4 = somewhat likely, 5 = very likely).

For hookah, blunts, and smoked marijuana and vaped marijuana assessed in Wave 3, participants answered the following question: How likely is it that over the next 6 months you will use [product]? Responses ranged from 1 to 5 (1 = already tried, 2 = very unlikely, 3 = somewhat unlikely, 4 = somewhat likely, 5 = very likely). The intention question was not asked for smokeless tobacco. These items were used as independent variables in bivariate correlation analysis and as comparators in predictive testing.

#### Perceived prevalence.

Participants in Wave 1 were asked, “Out of 100 teens your age, gender, and race/ethnicity, how many do you think use the products below [cigarettes, cigars, e-cigarettes, smokeless]?” which was responded to by filling in a number between 0 and 100. In Wave 3, participants filled in a number between 0 and 100 for the query: Out of 100 teens your age, how many do you think have used the products below [hookah, blunts, smoked- and vaped-marijuana] in the past 30 days?

#### Willingness.

In Wave 2 only, participants were asked: “If one of your friends were to offer you the following product, would you try it?” Response choices were: (1) definitely not, (2) probably not, (3) probably yes, and (4) definitely yes.

#### Behavior.

Participants were asked at all waves: “During your entire life how many times have you ever used [products]?” which was responded to as follows: “(1) never, (2) 1–2 times, (3) 3–10 times,(4) 11–19 times, (5) 20–30 times, (6) 31–99 times, and (7) 100 or more times.” For ease of interpretation, where appropriate, response categories were dichotomized into “never” (=1) and “ever” (>1).”

*Initiation/initiate* was computed as the change from never use of [product] (coded as 1) in Wave 1, 2, or 3 to any use of [product] (coded as 2–7) in any of Waves 5, 6, or 7.

*Escalation/escalate* was computed as the change in number of times used by ever-users (coded as 2, “1–2 times”) in Wave 1, 2, or 3 to any higher number of times used in any of Waves 5, 6, or 7.

### Analytic strategy.

We initially examined 26 Short-Term Risks, Benefits, and Long-Term Risks perceptions items and then discarded those with limited variation or too few participant responses. We formed candidate scales from the earliest of Waves 1 and 3 for which all measurement items for a given product were available. Measurements were taken from distinct samples, as indicated in tables; all significance tests were two-sided. Analyses were adjusted for clustering by school. Scales were correlated with measures from the same wave for convergent and discriminant validity analysis. When correlating scales with future behavior, comparable independent variables from the same wave were used where possible (i.e., with the willingness measure). Analysis was then carried out in three stages for each product using the final 19 Short-Term Risks and Benefits items.

First, to determine how many factors to extract for rotation, minimum average partials (MAP) was used (Velicer, 1976) for three reasons: (1) accuracy (84%), (2) tendency to under-factor when inaccurate (in 90% of inaccurate cases), and (3) factors with too small loadings were not retained (Hayton et al., 2004). As some data were missing, the expectation-maximization (EM) algorithm was used to estimate the variance/covariance matrix for each product and then that matrix was factor analyzed. This approach used all available data rather than excluding cases with a missing item. Oblique rotation was employed as it allows derived factors to correlate (Gable and Wolf, 2012). For ease of interpretation, rotated factor loading cutoffs of >0.40 were examined (Swisher et al., 2004; Tabachnick et al., 2007). The possible influence of missing data was examined by repeating the process using only records without missing data. The final factor loadings were virtually identical.

Items with weak or virtually no loadings were investigated as follows: first, descriptive statistics were computed, then correlation matrices including the problematic item and other items thought to be measuring the same construct were analyzed. Based on the strength of correlation and face validity of the item, a decision was made to either retain or remove the item. The seven Long-Term Risk items were not factored because they were highly correlated and with only seven items, we examined the correlation matrix manually and created a single scale by combining the items.

Second, once candidate scales were identified in the first step, Cronbach’s *α* was used to check internal consistency of the newly-created scales and to identify items that could be removed either because removal improved α or did not degrade *α* (dimension reduction). Cronbach’s *α* was recomputed for any changed scales. Thereafter, we checked the face validity of the scales and formed the final scales for all products. Scales were then correlated with each other and related independent variables to check for convergent and discriminant validity.

Third, we constructed correlation matrices to explore whether the newly-developed scales correlated with future behavior (i.e., initiation or escalation of use) and compared their predictive ability with related measures (i.e., general harm) and theoretical constructs shown to be predictive of behavior (i.e., willingness, perceived prevalence, and behavioral intention) (McEachan et al., 2011). All measures above were used as independent variables in bivariate correlation analysis and as comparators in predictive testing of initiation and escalation of tobacco and marijuana use. Age, a known correlate of perceptions and behavior, was adjusted for (Morrell et al., 2010; Song et al., 2009). We estimated Kendall’s tau-*b* correlation coefficients which are robust to non-linearity and extreme observations (Newson, 2002).

## Results

Our analysis of missing data and distribution resulted in 25 out of 26 potential specific perceptions items across products. The item “less hungry,” which correlated weakly with like items and did not load strongly on any factor across products, was removed from the analysis. The item “can quit” performed similarly but was necessarily retained in line with best practices for its function in the smokeless tobacco scale “addiction_risk” (Boateng et al., 2018). There were 22 indicators for hookah and smokeless; 21 for e-cigarettes; 20 each for cigars, blunts, and smoked marijuana; and 19 indicators each for cigarettes and vaped marijuana. Table 1 displays items included in created scales together with scale means, standard deviations, Cronbach’s *α*, and *N*.

### Factor analysis and internal consistency reliability.

Minimum average partials and factor analysis resulted in a five-factor solution for smokeless and a three-factor solution for the other seven products. The resulting factors (*F*) were named, based on items included in the final measurement scales, as follows: Long-Term Risks (F1), Short-Term Risks (F2), Benefits (F3); for smokeless, factors also included Social Risks (F4) and Addiction Risks (F5). Every item loaded above 0.46 on at least one factor except for “use in 5 years,” which loaded at 0.40 on F5. For all products except for smokeless, the underlying factors were labeled as: long-term risks, short-term risks, and benefits. When checked for internal consistency, the scales developed included two to 12 indicators and Cronbach’s *α* ranged from 0.69 to 0.96 (Table 1), demonstrating reasonable to good internal consistency (Cronbach, 1951; Nunnally and Bernstein, 1994).

### Convergent validity of the risks and benefits scales.

Correlation analysis among the factors indicated that scales representing risks (i.e., short-term risks, long-term risks, social risks, addiction risks) were highly interrelated and were not related to benefits. The long-term risks scale correlated positively with general harm for all products and with perceived prevalence for cigarettes, hookah, vaped marijuana, and blunts; long-term risks correlated negatively with behavioral intention for all products except cigars and e-cigarettes, with ever-use for all except hookah and smokeless, and with age for hookah, smoked marijuana and vaped marijuana, and blunts. The short-term risks scale correlated positively with general harm for all products and with perceived prevalence for cigarettes, vaped marijuana, blunts, and smokeless tobacco. The short-term risks scale correlated negatively with ever-use for all products except smokeless, with age for all except cigarettes, and with behavioral intention for all except cigars and e-cigarettes. The benefits scale correlated positively with perceived prevalence for all products, with ever-use for all products except smokeless, and with behavioral intention for all except cigars. Benefits correlated negatively with general harm for e-cigarettes, hookah, blunts, and smoked marijuana, it positively correlated); no correlation with age was found. The social risks scale correlated positively with general harm and negatively with age; the addiction risks scale correlated positively with general harm and perceived prevalence (Tables 2a and 2b).

### Analysis and comparison of predictive ability.

The variable or construct that most strongly predicted initiation for all products was willingness, followed by behavioral intention (except not for cigars and smokeless). The long-term risks scale and benefits scale were found to be third-most strongly predictive of initiation for different products. The long-term-risks scale was third for smokeless, e-cigarettes, blunts, and vaped marijuana and smoked marijuana and benefit came in third as predictors of initiation for all products except for e-cigarettes and smokeless. Fourth was the short-term risks scale (except for cigarettes and cigars). General harm predicted initiation for just three products (e-cigarettes and smoked marijuana and vaped marijuana), although the strength of the correlation was strong for both marijuana products. Perceived prevalence proved weakly predictive and only for initiation of blunts and smoked marijuana (Table 3).

Escalation of use for all products correlated strongly with willingness to use; other than the social risks scale that weakly predicted against escalation, willingness was the sole strong predictor for escalation of smokeless tobacco use. For hookah, blunts, and smoked marijuana and vaped marijuana, though, behavioral intention outperformed willingness as the strongest predictor of escalation. Our short-term risks scale was the third-best performer against escalation of hookah use, general harm was third (followed by long-term risks, fourth) for predicting against escalation of smoked marijuana. General harm was the next best (or fourth-best) performer against escalation of vaped-marijuana, followed by short-term risks (fifth). Perceived prevalence weakly predicted escalation of hookah, blunts, and smoked marijuana and vaped marijuana use, whereas general harm strongly predicted against *escalation* although only for smoked marijuana and vaped marijuana (Table 4).

## Discussion

In this study, we created and validated new scales that measure perceptions of specific risks and benefits associated with AYA use of cigarettes, cigars, smokeless tobacco, hookah, blunts, smoked marijuana, and vaped marijuana, and compared their predictive ability with measures of perceptions of general harm, social norms (perceived prevalence), willingness, and behavioral intentions. We report the following three key findings in answer to our research questions. First, measures of perceptions of specific social and health risks and benefits were less strongly associated than measures of perceptions of global outcomes with willingness, social norms, and intentions to use tobacco and marijuana; these differences varied by product. Scales measuring perceptions of specific risks (short-term, long-term, social, and addiction) were highly interrelated and not related to the benefits scales, which could indicate that AYA’s perceived risks and benefits are isolated or unrelated concepts. Second, although we demonstrated the reliability and validity of using the specific risk and benefit perceptions measurement scales to identify factors underlying motivation for initiation and escalation of tobacco and marijuana use, measures of perceived general harm outperformed specific measures in predicting uptake and continued use. Third, there were clear differences between the strength of correlation between specific measures of short-term and long-term risks and measures of willingness, social norms, and behavioral intentions, which likely indicates differences in utility and saliency of the scales for predicting behavior.

Study implications include support for the use of brief measures of perceived general harm when seeking to determine initiation and escalation of tobacco and marijuana use among AYA, resulting in shorter surveys. Shorter surveys reduce the risk and deleterious effects of participant fatigue from lengthy surveys containing a range of products and that enumerate specific risks and benefits, thereby garnering more accurate and useful data. In addition, the extent to which our scales of perceived health and social risks and benefits correlated differently across products suggests that these measures may capture motivational determinants that are product-focused. Further, such motivations may lie earlier in the causal chain and mediate actual use, which suggests that targeting these motivations may bring about behavior change (DiClemente et al., 2017; Michie and Johnston, 2012). For example, the perceived harmfulness of cigarettes appears to have permeated our sample, which could be due to tobacco control messaging, resulting in decreased reporting of use and of intentions to use. However, perceived short-term and long-term risks of using e-cigarettes, blunts, and smoked and vaped marijuana were not correlated with behavior or behavioral intentions, suggesting a need to address specific health and social outcomes perceptions.

Some of our findings relate to particular under-studied products and are therefore worth mentioning. For example, there are few longitudinal studies examining smokeless tobacco and AYA initiation and escalation in rural areas where such use is most widespread, and there is limited evidence of how perceptions of long-term risk influence smokeless tobacco use over time (Chaffee et al., 2019). It is plausible that the correlation we identified in this study between initiation of smokeless tobacco and the long-term risk scale could be due to long-standing social stigma related to smokeless tobacco, in addition to the scale’s specific delineated risks (Sami et al., 2012; Sterling and Mermelstein, 2011). Stigma is a likely factor in AYA decisions to use smokeless tobacco, especially when one considers that long-term health consequences of smokeless tobacco use (e.g., cancer, oral lesions, heart disease) are similar to long-term health consequences of using other tobacco products. Additionally, our finding that initiation of blunts most strongly correlated with the measure of perceived prevalence among peers comports with other studies reporting that initiation of blunts is most likely due to a perception that many peers are using them (M. L. Roditis et al., 2016). Ethnographic studies among adolescents and young adults also suggest that blunt use is more often perceived to have social benefits (Cole, 2006; Golub et al., 2005). These findings can guide development of future, in-depth studies that focus on the often-overlooked use of smokeless tobacco and burgeoning use of blunts among AYA.

All told, this study moves the needle on what is known about how measures of perceived general and specific risks and benefits correlate with each other as well as with measures of perceived prevalence, willingness, and behavioral intentions, and the comparative predictive utility of these constructs. Including a comprehensive range of conventional and newer tobacco and marijuana products in our study improves our understanding of AYA perceptions and motivations to use particular products. This study also paves the way for future work to look more closely at interactions between factors underlying theories of behavioral change and different tobacco and marijuana products. For example, social norms such as willingness to use substances and perceptions of prevalence most strongly predict initiating and/or escalating use of tobacco and marijuana, and this could plausibly be true for other drugs as well. Future studies may explore the extent of overlapping effects between risks, benefits, and social norms; such findings could provide a clearer understanding of AYA motivations and health behavior decision-making and could be used to address substance use in general. We also suggest measuring perceived likelihood of product-focused specific health and social outcomes to understand AYA motivations to use each tobacco and marijuana product.

### Limitations.

Data were collected prior to the e-cigarette or vaping product use-associated lung injury (EVALI) outbreak and the COVID-19 pandemic; if this study were conducted in present times, we may have found different perceptions of short-term and long-term risks of using e-cigarettes due to greater awareness of health-related harms of using these products among AYA. Our study did not examine whether marketing and advertising exposure and public health prevention messaging were additional factors influencing AYA perceptions of risk. While convergent validity of scales in our study reached statistical significance, most correlation coefficients were weak (<0.2); still, for Kendall’s tau-*b*, values of ≥0.15 are acceptable (Akoglu, 2018; Gilpin, 1993). While our specific measures were asked for other drugs, including alcohol, prescription drugs, and club- and harder-drugs, the number of participants reporting use was too small to support including them in our analyses. Still, our work here has paved the way to more easily discern generalizability of findings across the wide array of drugs used by AYA. While rates of product use aligned with those for California and the U.S. for the same time period of this study (Austin et al., 2018; Cullen et al., 2019; Glasser et al., 2019; McKelvey et al., 2018), our sample was about one year older than U.S. high-school students in general and rates may not reflect those in particular states in the U.S. or internationally. We acknowledge that the introduction of JUUL and other pod-based e-cigarettes to the U.S. market in the final waves of our longitudinal study may have influenced AYA initiation and escalation of e-cigarette products, and that the continually changing landscape of tobacco products will likely persist in altering perceptions about e-cigarette risks and benefits; thus, capturing nuances of motivation to use with our specific scales might be more useful than global measures in some instances.

## Conclusion

Measures of willingness and behavioral intentions together with measures of perceived general risks outperformed measures of specific outcomes such as short-term health and social risks, long-term risks, and benefits in predicting initiation and escalation of AYA tobacco and marijuana use. However, perceptions of specific health and social risks and benefits did correlate differently across tobacco and marijuana products, indicating a need for product-focused application of survey measures when trying to understand and differentiate across different substances.

## Figures and Tables

**Table 1:** Created scales by product with mean^a^ (SD), Cronbach’s *α*, and included items.

	Short-term risks									
	Health								Social^b^	Addiction^b^
	Cigarette (*n* = 569)	E-cigarette (*n* = 569)	Cigar (*n* = 568)	Smokeless (*n* = 567)	Hookah (*n* = 428)	Blunts (*n* = 428)	Vaped MJ (*n* = 430)	Smoked MJ (*n* = 429)	Smokeless (*n* = 561)	Smokeless (*n* = 552)
Mean (SD)	68.5 (18.6)	47.3 (26.5)	69.0 (20.9)	49.7 (26.9)	50.1 (19.2)	53.6 (18.6)	50.6 (19.4)	52.7 (19.1)	71.4 (22.0)	56.8 (20.7)
Cronbach’s *α*	0.89	0.92	0.88	0.86	0.87	0.88	0.88	0.88	0.78	0.69
Bad cough	X	X	X	X	X	N/A-	N/A	N/A	N/A	N/A
Worse sports	X	X	X	X	X	X	X	X	N/A	N/A
Catch breath	N/A	X	X	X	X	N/A	N/A	N/A	N/A	N/A
Cold	X	X	X	X	X	X	X	X	N/A	N/A
Feel high	N/A	X	X	X	X	N/A	N/A	N/A	N/A	N/A
In trouble	X	X	X	N/A	X	X	X	X	X	N/A
Friends upset	X	X	X	N/A	X	X	X	X	X	N/A
Mouth sores	X	X	X	X	X	X	X	X	N/A	N/A
Bad breath	X	X	X	N/A	X	X	X	X	X	N/A
Jittery	X	X	X	X	X	X	X	X	N/A	N/A
Use in 5 years	N/A	X	N/A	X	N/A	X	X	N/A	N/A	X
Get addicted	X	X	X	X	X	X	X	X	N/A	X
Quit anytime	N/A	N/A	N/A	X	N/A	N/A	N/A	N/A	N/A	X

	Long-term health risks							
	Cigarette (*n* = 534)	E-cigarette (*n* = 536)	Cigar (*n* = 534)	Smokeless (*n* = 535)	Hookah (*n* = 419)	Blunts (*n* = 420)	Vaped MJ (*n* = 422)	Smoked MJ (*n* = 420)
Mean (SD)	80.5 (19.8)	56.1 (31.9)	77.8 (20.7)	71.8 (23.4)	64.6 (26.6)	58.9 (28.9)	52.7 (30.3)	53.8 (30.2)
Cronbach’s *α*	0.91	0.96	0.91	0.90	0.92	0.93	0.92	0.92
Lung cancer	X	X	X	X	X	X	X	X
Tobacco death	X	X	X	X	X	X	X	X
Tobacco disease	X	X	X	X	X	X	X	X
Heart attack	X	X	X	X	X	X	X	X
Oral cancer	X	X	X	X	X	X	X	X
Wrinkles	X	X	X	X	X	N/A	N/A	N/A

	Benefits							
	Cigarette (*n* = 556)	E-cigarette (*n* = 556)	Cigar (*n* = 553)	Smokeless (*n* = 554)	Hookah (*n* = 425)	Blunts (*n* = 426)	Vaped MJ (*n* = 422)	Smoked MJ (*n* = 427)
Mean (SD)	21.8 (23.0)	19.0 (20.9)	20.6 (22.5)	16.3 (19.4)	20.6 (18.9)	23.0 (20.2)	16.3 (20.0)	23.2 (20.3)
Cronbach’s *α*	0.79	0.77	0.79	0.78	0.75	0.71	0.73	0.72
Less hungry	N/A	N/A	N/A	N/A	X	N/A	N/A	N/A
Concentrate	X	X	X	X	X	X	X	X
Less stressed	X	X	X	X	X	X	X	X
Look cool	X	X	X	X	X	X	X	X

a Percent chance of experiencing specific delineated outcomes for all scales; minimum is 0 and maximum is 100%.

b Social risks and Addiction risks were factors derived from items only for Smokeless.

**Table 2:** (a) Convergent validity—Kendall’s tau-*b* correlation coefficients for all products except smokeless. (b) Convergent Validity – Kendall’s tau-*b* Correlation Coefficients for Smokeless.

(a)							
	Benefits						
	Cigarette (*n* = 556)	Cigar (*n* = 553)	E-cigarette (*n* = 556)	Hookah (*n* = 424)	Smoked MJ (*n* = 426)	Vaped MJ (*n* = 427)	Blunts (*n* = 425)
General harm^a^	−0.02	−0.04	**−0.13**	**−0.14**	**−0.15**	−0.08	**−0.15**
Intention^b^	**0.11**	0.04	**0.09**	**0.15**	**0.28**	**0.16**	**0.28**
Perceived prevalence^c^	**0.17**	**0.18**	**0.22**	**0.25**	**0.25**	**0.27**	**0.27**
Ever-use^d^	**0.15**	**0.08**	**0.11**	**0.16**	**0.21**	**0.13**	**0.23**
Age^e^	0.02	0.01	−0.05	0.05	0.05	0.05	0.04
**Short-term risks**							
	**Cigarette (*n* = 569)**	**Cigar (*n* = 568)**	**E-cigarette (*n* = 569)**	**Hookah (*n* = 427)**	**Smoked MJ (*n* = 428)**	**Vaped MJ (*n* = 429)**	**Blunts (*n* = 427)**
General harm^a^	**0.33**	**0.34**	**0.60**	**0.32**	**0.46**	**0.47**	**0.42**
Intention^b^	**0.08**	−0.004	0.05	**−0.27**	**−0.30**	**−0.23**	**−0.23**
Perceived prevalence^c^	**0.12**	0.05	−0.06	0.05	−0.01	**0.12**	**0.04**
Ever-use^d^	**0.09**	**−0.12**	**−0.31**	**−0.19**	**−0.25**	**−0.19**	**−0.19**
Age^e^	−0.06	**−0.10**	**−0.17**	**−0.18**	**−0.22**	**−0.19**	**−0.20**
**Long-term risks**							
	**Cigarette (*n* = 534)**	**Cigar (*n* = 534)**	**E-cigarette (*n* = 536)**	**Hookah (*n* = 417)**	**Smoked MJ (*n* = 418)**	**Vaped MJ (*n* = 420)**	**Blunts (*n* = 418)**
General harm^a^	**0.28**	**0.25**	**0.56**	**0.19**	**0.55**	**0.53**	**0.47**
Intention^b^	**−0.13**	−0.07	0.04	**−0.13**	**−0.32**	**−0.28**	**−0.26**
Perceived prevalence^c^	**0.11**	0.07	0.03	**0.15**	−0.02	**0.11**	**0.02**
Ever-use^d^	**−0.15**	**−0.11**	**−0.27**	−0.09	**−0.31**	**−0.23**	**−0.22**
Age^e^	−0.06	−0.07	−0.08	**−0.11**	**−0.20**	**−0.18**	**−0.19**

(b)					
	Smokeless benefit (*n* = 554)	Smokeless short-term risks			(*n* = 535)
		Health (*n* = 567)	Social (*n* = 561)	Addiction (*n* = 552)	
**smokeless long-term risks**					
General harm^a^	−0.07	**0.37**	**0.35**	**0.17**	**0.40**
Perceived prevalence^c^	**0.23**	**0.08**	0.02	**0.18**	0.06
Ever-use^d^	−0.04	−0.07	−0.07	0.02	−0.06
Age^e^	−0.02	**−0.14**	**−0.11**	0.03	−0.06

*Note*: Boldface correlation coefficients indicate statistical significance at *p* < 0.05.

**Table 3:** Kendall’s tau-*b* correlation coefficients^a^ for INITIATE.

	Short-term risks	Long-term risks	General harm	Benefits	Perceived prevalence	Intention	Willingness
Cigarettes (*n* = 565)				0.07732		0.09393	0.24483
Cigars (*n* = 565)				0.08871			0.25987
E-cigarettes (*n* = 565)	−0.12356	−0.10296	−0.16332			0.11141	0.44097
Smokeless (*n* = 565)	−0.08322	−0.13061					0.23606
Hookah (*n* = 508)	−0.16015			0.08805		0.26391	0.37097
Blunts (*n* = 508)	−0.15346	−0.14379		0.08902	0.15414	0.37697	0.43064
Smoked_MJ (*n* = 508)	−0.13875	−0.11029	−0.22823	0.18035	0.16521	0.46407	0.40447
Vaped_MJ (*n* = 508)	−0.18846	−0.15243	−0.22192	0.09454		0.33782	0.32026

a *Note*: All correlation coefficients reflected in this table are significant at *p* < 0.05.

**Table 4:** Kendall’s tau-*b* correlation coefficients^a^ for ESCALATE.

	Short-term risks	Long-term risks	Social risks	General harm	Benefits	Perceived prevalence	Intention	Willingness
Cigarettes					0.0903		–	0.30686
Cigars							–	0.3217
E-cigarettes	−0.08635	−0.09359			0.11969		–	0.35817
Smokeless			−0.09033				–	0.29572
Hookah	−0.15548	−0.08986			0.12224	0.12967	0.34944	0.39913
Blunts	−0.16632	−0.13849			0.21949	0.12561	0.50732	0.40559
Smoked_MJ	−0.16158	−0.19328		−0.26435	0.15975	0.13748	0.48664	0.45759
Vaped_MJ	−0.18254	−0.16195		−0.22881	0.09867	0.08184	0.42215	0.37201

a *Note*: All correlation coefficients reflected in this table are significant at *p* < 0.05.

– Not calculated.
